# 1284. Racial disparities in periprosthetic joint infections: A retrospective study

**DOI:** 10.1093/ofid/ofad500.1123

**Published:** 2023-11-27

**Authors:** Joshua B Davis, Matthew P Jamison, Adriana Liimakka, Antonia Chen, Sandra B Nelson, Jodian Pinkney

**Affiliations:** Brigham and Women's Hospital, Boston, Massachusetts; Brigham and Women's Hospital, Boston, Massachusetts; Harvard Medical School, Boston, Massachusetts; Brigham and Women's Hospital, Boston, Massachusetts; Massachusetts General Hospital, Boston, MA; Massachusetts General Hospital , Boston, Massachusetts

## Abstract

**Background:**

Total joint arthroplasty (TJA) is one of the most common surgical operations performed in the US. Periprosthetic joint infection (PJI) is a daunting complication of TJA and is associated with high morbidity. Previous studies have shown that racial disparities exist in postoperative complications of TJAs, including readmissions and mortality. However, it is unclear if such disparities also exist for PJI.

**Methods:**

A single large hospital system database was used to identify all patients who underwent either primary total knee arthroplasty (TKA) or total hip arthroplasty (THA) between January 2018 and December 2021. Patients were stratified by self-identified racial categories. PJI was defined by ICD-9/10 coding. We present descriptive data as frequencies and percentages for categorical variables and means and standard deviations for continuous variables. We used chi-square tests to estimate crude risk ratios (cRR) and 95% confidence intervals (CIs) for PJI by race.

**Results:**

A total of 11,818 patients were included in the final analysis. The majority (96.6%) of patients identified as non-Black (Table 1). The mean age was 69.4 (±10.3) years for non-Black patients and 65.5 (±11.5) years for Black patients. Females represented the majority of non-Black and Black patients who underwent TJA (54.1% and 66.9%, respectively). The majority of TJAs were TKAs for both non-Black and Black patients (54.6% and 56.1%, respectively). The incidence of PJIs was 1.6% among non-Black patients compared to 3.3% among Black patients (cRR (95% CI): 1.99 (1.17 – 3.39); p=0.0112). The majority of PJIs occurred post TKA (Table 1).

**Figure:**
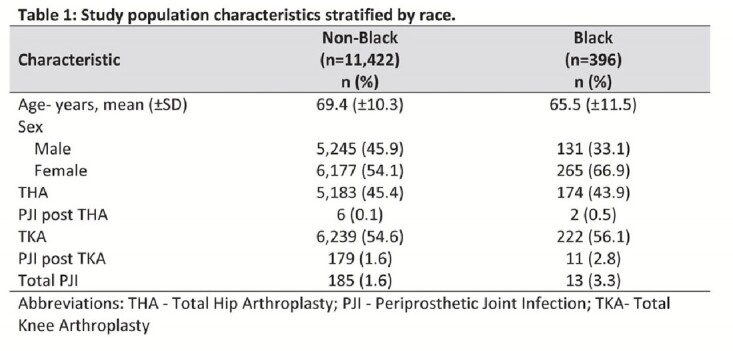

**Conclusion:**

Preliminary findings suggest that racial disparities exist in PJIs. These findings were not adjusted for possible confounding factors such as age, sex and comorbidities. Future studies are needed to determine if such disparities persist when other factors are considered.

**Disclosures:**

**Antonia Chen, MD, MBA**, Adaptive Phage Therapeutics: Advisor/Consultant|Adaptive Phage Therapeutics: Grant/Research Support|Avanos: Advisor/Consultant|BICMD: Advisor/Consultant|Convatec: Advisor/Consultant|Elute: Grant/Research Support|Ethicon: Advisor/Consultant|GLG: Advisor/Consultant|Guidepoint: Advisor/Consultant|Heraeus: Advisor/Consultant|Hyalex: Stocks/Bonds|IlluminOss: Stocks/Bonds|Irrimax: Advisor/Consultant|Irrimax: Stocks/Bonds|Journal of Bone and Joint Surgery: Deputy Editor|Osteal Therapeutics: Advisor/Consultant|Osteal Therapeutics: Stocks/Bonds|Peptilogics: Advisor/Consultant|Peptilogics: Grant/Research Support|Pfizer: Advisor/Consultant|SLACK Incorporated: royalties|Smith and Nephew: Advisor/Consultant|Sonoran: Stocks/Bonds|Stryker: Advisor/Consultant|Taylor & Francis Group: Royalties|UpToDate: Royalties

